# The Perfect Storm - A Bio-Psycho-Social Risk Model for Developing and Maintaining Eating Disorders

**DOI:** 10.3389/fnbeh.2016.00044

**Published:** 2016-03-10

**Authors:** Guido K. W. Frank

**Affiliations:** Departments of Psychiatry and Neuroscience, Children's Hospital Colorado, University of Colorado Anschutz Medical CampusAurora, CO, USA

**Keywords:** anorexia, bulimia, eating disorders, model, development, psychopathology, trait, risk

The eating disorders (EDs), anorexia nervosa (AN), and bulimia nervosa (BN) are severe psychiatric disorders of unknown etiology. EDs usually begin during adolescence and occur most commonly in females (American Psychiatric Association, 2013). The diagnostic criteria for AN include restriction of energy intake leading to significantly low body weight, intense fear of gaining weight, or persistent behaviors that interfere with weight gain, and disturbance in the way one's body weight, or shape is experienced (American Psychiatric Association, 2013). A restricting type (AN-R), marked by food restriction and commonly over-exercising, has been distinguished from a binge-eating/purging type (AN-B/P), where afflicted individuals eat large amounts of food in a relatively short period of time (“binge eating”), or engage in behaviors to counteract weight gain, such as self-induced vomiting or use of laxatives, or diuretics (“purging”). BN individuals are usually at normal weight, and engage in recurrent binge eating and purging behavior at least once a week for at least 3 months. A new diagnosis, “binge eating disorder” (BED) is part of the ED diagnostic categories in DSM-5. BED is associated with regular binge eating episodes without compensatory mechanisms. Individuals with ED symptoms that do not meet full criteria for AN or BN were classified as ED not otherwise specified (NOS) in the past but now fall into the categories of “other specified feeding or eating disorders (OSFED)” or “unspecified feeding or eating disorder (UFED; American Psychiatric Association, 2013).”

A variety of risk factors have been identified in the past that could predispose an individual to developing an ED, as most recently and extensively reviewed by Culbert et al. (2015). Sociocultural expectations of thinness are a risk factor for all EDs (Keel and Forney, 2013); with respect to personality traits, negative emotionality, perfectionism, and negative urgency (tendency to engage in rash action when distressed) have been associated with ED development (Racine et al., 2013; Boone et al., 2014); there is evidence that the brain neurotransmitters serotonin and dopamine are associated with AN and BN, altered gene expression (epigenetics) also appears to be part of the group of risk factors for AN and BN, and increased hormone release during puberty and its effect on reward processing could contribute to the typical age of onset during adolescence. What has received little attention in the literature is how biological factors *per-se* not only could predispose an individual to develop an eating disorder, but how the interaction of a potential neurotransmitter trait vulnerability and particular eating behavior could change brain biology and create risk for chronic illness.

In this article I propose that extremes of eating in human EDs alter brain function, in particular dopamine related pathways, and create a biological vicious cycle that interferes with recovery.

The complex interactions between psychosocial and neurobiological abnormalities in EDs have limited the development of neuroscience-based treatments (Agras et al., 2004; Kaye et al., 2013). It is possible that (a) state of illness and severity of ED symptoms are associated with degrees of alterations of brain function (Kaye et al., 2013), and (b) different ED characteristics could be related to distinct neurobiological abnormalities that contribute to overlap in symptoms across EDs. However, no comprehensive biological models exist to supplement the sociocultural factors and personality traits based models that describe the development of ED pathophysiology across the whole spectrum, and that can be tested empirically. ED characteristics with a focus on binge/purge behaviors, being over- or underweight or traits such as sensitivity to salient stimuli (Jappe et al., 2011) could for instance be tied to specific brain alterations independent from categorical diagnostic boundaries. This approach could be successful in understanding the underlying neurobiology, categorizing EDs based on brain function-behavior relationships, and produce individualized treatments. This has not been specifically explored before with respect to biological underpinnings. Such “dimensional” research in the ED population based on specific ED behaviors across traditional diagnostic categories could significantly advance our ability to connect ED behaviors with specific brain mechanisms. Furthermore, this would help us identify how cortical higher order cognitive emotional processes interact with subcortical, developmentally “older,” brain structures that drive behaviors such as eating on a more basic stimulus-response level. Such an advanced understanding of ED pathophysiology may be necessary in order to understand the neurobiology of EDs in general and enable us to model patient-specific brain alterations, in order to develop individualized and more effective treatments.

Some research has suggested dimensions of altered brain function across the spectrum of disordered eating. For instance brain research across AN, BN, and obesity has shown that being underweight is associated with heightened, while being overweight is associated with reduced brain response, in the insula, the subcortical ventral striatum, and the prefrontal cortex (Frank et al., 2012). That study used a dopamine anchored brain reward model using taste stimuli as well as learning and prediction of those stimuli and the results suggested that dopamine response could be responsible for the results found, as it mirrored results form basic science research (Frank et al., 2012). In addition, the more often a person with BN binged and purged, the lower this person's brain response was (Frank et al., 2011). Thus, there appear to be very specific alterations in response to extremes or food intake in taste and subcortical dopamine related reward circuits (Johnson and Kenny, 2010).

On the other hand, ED behaviors are highly driven by the well-known fear of having too high of a body weight or not fitting a self-perceived societal expectation of thinness (Keel and Forney, 2013). Those fears often develop after negative feedback from the environment. Individuals with an ED tend to be unhappy with themselves but the ED enhances a sense of control and happiness, and reduces anxiety (Dignon et al., 2006). Individuals who develop an ED frequently already had premorbid high anxiety, which creates a sense of uncertainty, making them more vulnerable to negatively perceived feedback from the environment (Kaye et al., 2004). Perfectionism and negative urgency predict the development of eating disorder symptoms, however, the specific mechanisms and underlying biological factors are unclear (Racine et al., 2013; Boone et al., 2014). In summary, there is a host of factors that have to come together in order for a person to develop an ED although our knowledge of the underlying mechanisms is limited.

Using the above information and taking into account potential bio-psycho-social developmental risk factors for developing EDs, I propose a model that demonstrates how EDs may be a culmination of those factors and creating a “perfect storm” and where changes in brain dopamine function have an important role; this is outlined in Figure 1. However, the dopamine system is for sure only one aspect of brain neurotransmitter function that may be affected by disordered eating (Manuel-Apolinar et al., 2014):

1. It is possible that individuals who will develop an ED are born with biological traits such as disposition to heightened anxiety (Kaye et al., 2004), increased sensitivity to salient stimuli (Jappe et al., 2011) as well as a larger (AN, BN) or smaller (BED, obesity) orbitofrontal cortex, which could alter the individual's ability to stop eating (Frank et al., 2013a,b). In addition, it could be that EDs are associated with dopamine circuits that are less stable during development and might adapt too quickly to environmental influences. For instance, individuals who develop AN may be particularly sensitive to times when there is less food eaten, such as skipping meals when having a busy schedule, voluntary fasting for religious reasons, or changing eating behavior in an attempt to eat “healthier,” which frequently occurs in middle school after health class. In those individuals the dopamine system might get overly sensitized to a point where stimulation is perceived as excessive as opposed to driving healthy eating. This together with anxiety and high cognitive control as typically seen in AN may lay the foundation for the disorder risk. BN has also been associated with high cognitive control, anxiety, and sensitivity to salient stimuli, but they may be more sensitive to excessive food intake and desensitization of dopamine terminals. While individuals with BN often restrict food intake especially during the day, they also have times when they “have to give in” to the urge to binge and purge, and the desensitized dopamine system overrides the cognitive control and needs stimulation. We know little about BED brain function, but based on our studies in obesity one could hypothesize that in this group dopamine circuits are easily desensitized to excessive food, and that paired with a less strong cognitive control mechanism could predispose an individual to have difficulty stopping eating, even when physiologically sufficient food has been eaten (Frank et al., 2012). Taken together, there may be underlying traits, maybe on opposite ends that correspond to the phenotypically opposing eating disorder behaviors that create vulnerability. Under certain environmental conditions, those traits then may become relevant and contribute to illness development.
2. Specific environmental triggers then may set off active illness behavior. Such triggers may include either states of food restriction (travel, camp, busy schedule) or excessive food intake (holidays, frequently being exposed to buffet meals, familial habit to present large portions), which may interact with dopamine circuits that are vulnerable to the effects of high or low food intake. Another important factor in triggering dopamine system alterations is puberty. Estrogen has a variety effects on eating behavior via neurotransmitter function and may play an important role in ED risk (Asarian and Geary, 2013). With menarche there is a surge in gonadal hormones including estrogen, which has profound effects on dopamine release (Garris and Ben-Jonathan, 1991; Alfinito et al., 2009). EDs typically begin during the years of sexual maturation and around menarche, and estrogen—dopamine interaction could contribute mechanistically to their development. This change in estrogen mediated dopamine release could add to over-stimulation in AN promoting aversive response to salient stimuli, but a dopamine system vulnerable to desensitization could down-regulate too strongly in response to the strong physiological dopamine release.
3. Well-known factors such as problems in family functioning, poor self-esteem, and social pressure may drive altered food intake in response to those difficult situations that create negative emotions and heightened stress. Stress neurobiologically alters dopamine and opioid neurotransmission (Latagliata et al., 2014) and phenotypically affects eating behavior (Harris, 2015). Stress, therefore could have a crucial mechanistic role in driving a person into eating disorder behaviors. The social pressure to be thin also creates stress but also frequently leads to positive feedback (psychological reward) from the environment after weight loss. This promotes more weight loss and potentially dopamine sensitization, but also stress because of increased fear of weight gain. On the other hand, weight gain in BN or BED is frequently associated with negative feedback, this typically leads to lower mood, and eating is often used to handle stress from the sense of social inferiority.
4. In the last step, a perpetuating vicious cycle develops. Food restriction in AN reduces overstimulation but further sensitizes the dopamine system. That together with high fear of weight gain then creates a situation where the normal eating stimulating mechanisms fail and dangerous weight loss ensues. In BN, the high cognitive control paired with hyposensitive dopamine circuits may lead to episodic food restriction interrupted by binge eating and purging episodes. In obesity and maybe also BED, high food intake further desensitizes dopaminergic circuits, resulting in even more food intake in order to satisfy the need for stimulation of this circuitry.

**Figure 1:**
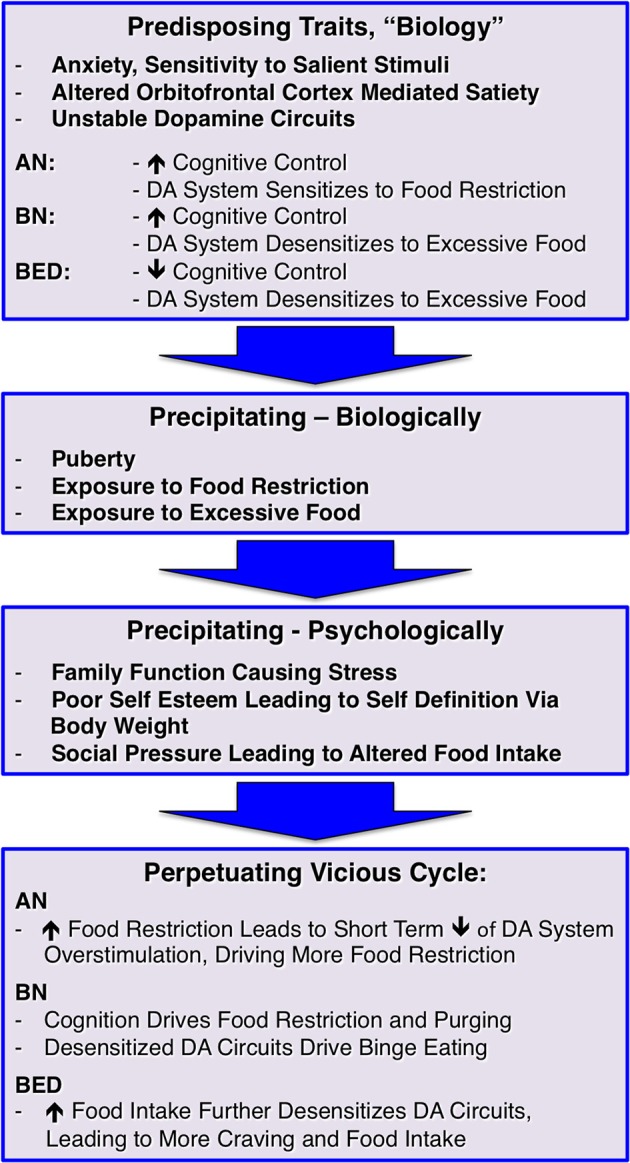
**A bio-psycho-social risk model for developing and maintaining eating disorders; dopamine, DA**.

EDs are highly complex problems and we do not have good models how bio-psycho-social factors interact to result in developing an ED. Here, I provided a potential framework for future research and testing of hypotheses what biological mechanisms may underlie those disorders. This model does not assume to be complete and may rather be a starting point for development of more complex descriptions of what risk factors may have to come together to drive dangerously altered eating behavior.

## Author contributions

The author confirms being the sole contributor of this work and approved it for publication.

## Funding

This work was supported by NIMH grant R01 MH096777, NIMH grant R01 MH103436, and by the Davis Foundation Award of the Klarman Family Foundation Grants Program in Eating Disorders (all GF).

### Conflict of interest statement

The author declares that the research was conducted in the absence of any commercial or financial relationships that could be construed as a potential conflict of interest.

## References

[B1] Agras W. S. Brandt H. A. Bulik C. M. Dolan-Sewell R. Fairburn C. G. Halmi K. A.. (2004). Report of the National Institutes of Health workshop on overcoming barriers to treatment research in anorexia nervosa. Int. J. Eat. Disord. 35, 509–521. 10.1002/eat.10261 15101067

[B2] Alfinito P. D. Chen X. Mastroeni R. Pawlyk A. C. Deecher D. C. (2009). Estradiol increases catecholamine levels in the hypothalamus of ovariectomized rats during the dark-phase. Eur. J. Pharmacol. 616, 334–339. 10.1016/j.ejphar.2009.06.045 19576879

[B3] American Psychiatric Association (2013). Diagnostic and Statistical Manual of Mental Disorders, 5th Edn (DSM-5(TM)). Arlington, VA: American Psychiatric Publishing.

[B4] Asarian L. Geary N. (2013). Sex differences in the physiology of eating. Am. J. Physiol. Regul. Integr. Comp. Physiol. 305, R1215–R1267. 10.1152/ajpregu.00446.2012 23904103 PMC3882560

[B5] Boone L. Soenens B. Luyten P. (2014). When or why does perfectionism translate into eating disorder pathology? A longitudinal examination of the moderating and mediating role of body dissatisfaction. J. Abnorm. Psychol. 123, 412–418. 10.1037/a0036254 24886015

[B6] Culbert K. M. Racine S. E. Klump K. L. (2015). Research review: what we have learned about the causes of eating disorders - a synthesis of sociocultural, psychological, and biological research. J. Child Psychol. Psychiatry 56, 1141–1164. 10.1111/jcpp.12441 26095891

[B7] Dignon A. Beardsmore A. Spain S. Kuan A. (2006). ‘Why I won't eat’: patient testimony from 15 anorexics concerning the causes of their disorder. J. Health Psychol. 11, 942–956. 10.1177/1359105306069097 17035265

[B8] Frank G. K. Reynolds J. R. Shott M. E. Jappe L. Yang T. T. Tregellas J. R.. (2012). Anorexia nervosa and obesity are associated with opposite brain reward response. Neuropsychopharmacology 37, 2031–2046. 10.1038/npp.2012.51 22549118 PMC3398719

[B9] Frank G. K. Reynolds J. R. Shott M. E. O'Reilly R. C. (2011). Altered temporal difference learning in bulimia nervosa. Biol. Psychiatry 70, 728–735. 10.1016/j.biopsych.2011.05.011 21718969 PMC3186835

[B10] Frank G. K. Shott M. E. Hagman J. O. Mittal V. A. (2013a). Alterations in brain structures related to taste reward circuitry in ill and recovered anorexia nervosa and in bulimia nervosa. Am. J. Psychiatry 170, 1152–1160. 10.1176/appi.ajp.2013.12101294 23680873 PMC3789862

[B11] Frank G. K. Shott M. E. Hagman J. O. Yang T. T. (2013b). Localized brain volume and white matter integrity alterations in adolescent anorexia nervosa. J. Am. Acad. Child Adolesc. Psychiatry 52, 1066–1075.e1065. 10.1016/j.jaac.2013.07.007 24074473 PMC4082770

[B12] Garris P. A. Ben-Jonathan N. (1991). Estradiol rapidly stimulates dopamine release from the posterior pituitary *in vitro*. Neuroendocrinology 53, 601–607. 10.1159/000125780 1876237

[B13] Harris R. B. (2015). Chronic and acute effects of stress on energy balance: are there appropriate animal models? Am. J. Physiol. Regul. Integr. Comp. Physiol. 308, R250–R265. 10.1152/ajpregu.00361.2014 25519732 PMC4329465

[B14] Jappe L. M. Frank G. K. Shott M. E. Rollin M. D. Pryor T. Hagman J. O.. (2011). Heightened sensitivity to reward and punishment in anorexia nervosa. Int. J. Eat. Disord. 44, 317–324. 10.1002/eat.20815 21472750 PMC3072848

[B15] Johnson P. M. Kenny P. J. (2010). Dopamine D2 receptors in addiction-like reward dysfunction and compulsive eating in obese rats. Nat. Neurosci. 13, 635–641. 10.1038/nn.2519 20348917 PMC2947358

[B16] Kaye W. H. Bulik C. M. Thornton L. Barbarich N. Masters K. (2004). Comorbidity of anxiety disorders with anorexia and bulimia nervosa. Am. J. Psychiatry 161, 2215–2221. 10.1176/appi.ajp.161.12.2215 15569892

[B17] Kaye W. H. Wierenga C. E. Bailer U. F. Simmons A. N. Bischoff-Grethe A. (2013). Nothing tastes as good as skinny feels: the neurobiology of anorexia nervosa. Trends Neurosci. 36, 110–120. 10.1016/j.tins.2013.01.003 23333342 PMC3880159

[B18] Keel P. K. Forney K. J. (2013). Psychosocial risk factors for eating disorders. Int. J. Eat. Disord. 46, 433–439. 10.1002/eat.22094 23658086

[B19] Latagliata E. C. Valzania A. Pascucci T. Campus P. Cabib S. Puglisi-Allegra S. (2014). Stress-induced activation of ventral tegmental mu-opioid receptors reduces accumbens dopamine tone by enhancing dopamine transmission in the medial pre-frontal cortex. Psychopharmacology (Berl). 231, 4099–4108. 10.1007/s00213-014-3549-7 24958228

[B20] Manuel-Apolinar L. Rocha L. Damasio L. Tesoro-Cruz E. Zarate A. (2014). Role of prenatal undernutrition in the expression of serotonin, dopamine and leptin receptors in adult mice: implications of food intake. Mol. Med. Rep. 9, 407–412. 10.3892/mmr.2013.1853 24337628 PMC3896523

[B21] Racine S. E. Keel P. K. Burt S. A. Sisk C. L. Neale M. Boker S.. (2013). Exploring the relationship between negative urgency and dysregulated eating: etiologic associations and the role of negative affect. J. Abnorm. Psychol. 122, 433–444. 10.1037/a0031250 23356217 PMC3759363

